# Paeonol as a Multi‐Target Therapy for Neurological Disorders: Mechanistic Insights Into Neuroprotection

**DOI:** 10.1002/iid3.70494

**Published:** 2026-08-17

**Authors:** Md. Sakib Al Hasan, Naznin Shahria, Yasin Emon, Mohammad Y. Alshahrani, Emon Mia, Faisal H. Altemani, Rituparna Biswas Suma, Umme Habiba Sumaya, Mohammed Burhan Uddin, Md. Torequl Islam

**Affiliations:** ^1^ Department of Pharmacy Gopalganj Science and Technology University Gopalganj Bangladesh; ^2^ Department of Pharmacy Khwaja Yunus Ali University Enayetpur Sirajganj Bangladesh; ^3^ Department of Pharmacy Islamic University Kushtia Bangladesh; ^4^ Central Labs King Khalid University Abha Saudi Arabia; ^5^ Department of Clinical Laboratory Sciences, College of Applied Medical Sciences King Khalid University Abha Saudi Arabia; ^6^ Department of Medical Laboratory Technology, Faculty of Applied Medical Sciences University of Tabuk Tabuk Saudi Arabia; ^7^ Department of Chemistry York College of the City University of New York Jamaica New York United States; ^8^ Phytochemistry and Biodiversity Research Laboratory BioLuster Research Center Ltd Gopalganj Bangladesh; ^9^ Centre for Research Development University of Cyberjaya Cyberjaya Malaysia

**Keywords:** Alzheimer's disease, neuroinflammation, neuroprotection, Paeonol, Parkinson's disease

## Abstract

**Background:**

Neurological disorders are a major global health challenge in the current era, and they need new therapeutic treatments.

**Aims:**

This review evaluates the neurobiological activity of Paeonol (PNL) focusing on its antioxidant, anti‐inflammatory, and cognitive enhancing effects in various neurodegenerative disorders.

**Methods:**

A literature search (up to March 2025) was conducted via PubMed, ScienceDirect, Scopus, and other databases using MeSH terms related to PNL's pharmacology and neuroprotection. Inclusion criteria encompassed preclinical studies (in vitro and in vivo) and clinical trials, while exclusion criteria eliminated non‐English papers and non‐neurodegenerative research.

**Results:**

PNL, a bioactive phenolic compound from *Paeonia suffruticosa*, has shown antioxidative, anti‐inflammatory, and neuroprotective effects. PNL reduces oxidative stress by boosting the activity of superoxide dismutase (SOD) and glutathione (GSH) while decreasing reactive oxygen species (ROS) and lipid peroxidation. It also reduces neuroinflammation by inhibiting key pathways such as nuclear factor kappa‐light‐chain‐enhancer of activated B cells (NF‐κB), toll‐like receptor 4 (TLR4), and mitogen‐activated protein kinase (MAPK), and it affects apoptosis‐related proteins like B‐cell lymphoma 2 (Bcl‐2) and Bcl‐2‐associated X protein (Bax). In Alzheimer's disease (AD) models, PNL reduces amyloid‐β plaques and some pro‐inflammatory cytokines like interleukin‐1 beta (IL‐1β) and tumor necrosis factor‐alpha (TNF‐α), improving cognitive function. For ischemic stroke, it reduces infarction volume and microglial activation by targeting TLR2/4 and NF‐κB. PNL has anticonvulsant effects in epilepsy and protects dopaminergic neurons in Parkinson's disease (PD). It also demonstrates anxiolytic and antidepressant properties by modulating Brain‐Derived Neurotrophic Factor (BDNF), Nerve Growth Factor (NGF), and oxidative stress markers. PNL is quickly absorbed with moderate bioavailability and low blood–brain barrier permeability. It effectively treats unstable angina but is less effective for osteoarthritis pain. Though safe at therapeutic doses, high concentrations can be cytotoxic.

**Conclusion:**

Further clinical research is needed to confirm its neurotherapeutic potential.

## Graphical abstract

Paeonol (PNL) exhibits neuroprotective effects by reducing oxidative stress, neuroinflammation, and apoptosis. It enhances antioxidant defenses (SOD and GSH), inhibits pro‐inflammatory pathways (NF‐κB and TLR4), and modulates apoptotic proteins (Bcl‐2 and Bax). PNL improves cognitive and motor functions in neurodegenerative disorders, epilepsy, and stroke, highlighting its therapeutic potential.

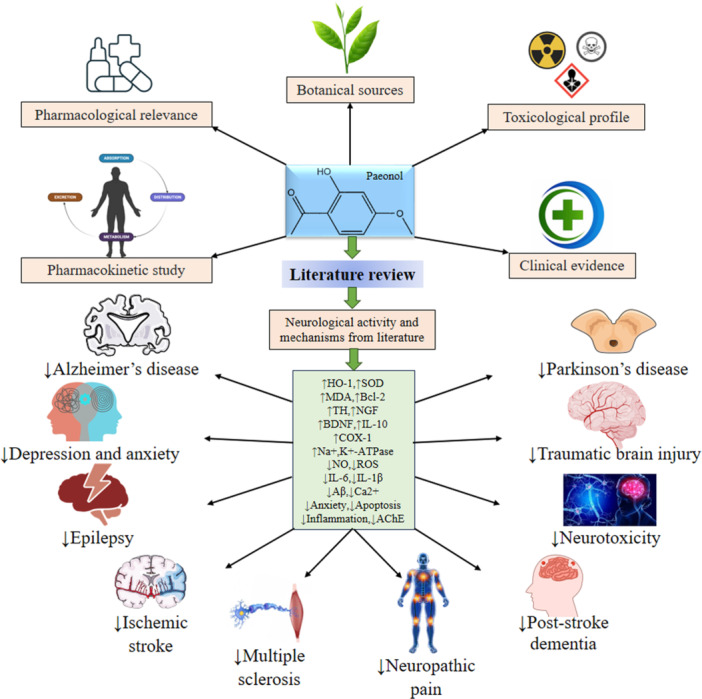

## 1 Introduction

Diseases of the neurological system that affect the brain and spinal cord are often thought of as age‐related, incurable, and basically untreatable [1]. A significant percentage of the global disease burden is caused by neurological disorders, which constitute a serious and growing global health concern. Nearly 10 million fatalities and 349 million DALYs worldwide were attributed to neurological illnesses in 2019 [2].

Natural products are bioactive compounds derived from plants, animals, or microorganisms that serve as a major source of drug discovery [3, 4, 5]. They offer unique chemical diversity and biological activities that inspire new therapeutic agents [6, 7]. Their antioxidant, anti‐inflammatory, and neuroprotective properties make them promising candidates for treating neurological disorders [8]. Many natural compounds can modulate neurotransmitters and protect neurons from oxidative damage [9, 10]. Thus, they provide valuable leads for developing safe and effective neuroprotective drugs. Phytoconstituents like polyphenolic antioxidants, which are available in marine flora and freshwater, in addition to herbs, nuts, fruits, and vegetables may be able to prevent neurodegeneration and enhance memory and cognitive processes in the brain. They are also recognized to be essential in the prevention and treatment of various neurodegenerative illnesses, including PD, AD, epilepsy, and other neurological conditions [8]. Vitamin E and C and beta‐carotene are examples of natural antioxidants that can be used to scavenge free radicals produced as Alzheimer's disease progresses [11]. Another study by Siddiqui et al. (2024) reviewed the therapeutic potential of baicalein, a flavonoid with strong antioxidant, anti‐inflammatory, and neuroprotective properties. The study highlights its effectiveness in managing Alzheimer's, Parkinson's, and other neurodegenerative diseases. It also discusses molecular mechanisms and recent advances supporting baicalein's role in neuroprotection [12].

PNL with molecular formula C_9_H_10_O_3_ (1‐(2‐hydroxy‐4‐methoxyphenyl) ethanone) is obtained from *Paeonia suffruticosa* and *P. lactiflora*. PNL shows antioxidative and anti‐inflammatory activity that can reduce the volume of cerebral infarction and alleviate neurological impairments [13]. PNL reduced APP, BACE, and apoptosis to protect memory following an ischemic stroke [14]. PNL strongly activates the GSK3α/β and AMPKα signaling pathways to prevent oxidative and inflammatory mediators. PNL markedly reduced the production of COX‐2 and iNOS, as well as the emission of NO in neurodegenerative disease. Additionally, PNL treatment lessened the generation of ROS and prevented an increase in cell migration brought on by ATP [15]. In PTZ‐induced seizures, PNL has anticonvulsant and neuroprotective properties. The suppression of oxidative stress pathways may be linked to these outcomes [16].

This review investigates the neuroprotective potential of PNL by analyzing its antioxidative, anti‐inflammatory, and anti‐apoptotic mechanisms in various neurological disorders alongside reviewing the pharmacokinetic limitations and clinical translation challenges, and proposes strategies to enhance therapeutic efficacy and safety for neurological disorders.

## 2 Methodology

### 2.1 Literature Search Strategy

As of March 25, 2025, a comprehensive literature search was done to evaluate the pharmacological and neuroprotective properties of PNL. The search was performed across multiple scientific databases, including Google Scholar, PubMed/Medline, ScienceDirect, Scopus, Wiley Online Library, SpringerLink, Web of Science, and various patent offices (such as USPTO, CIPO, and WIPO) using the following MeSH terms: “PNL/pharmacology,” “PNL/administration & dosage,” “Neurodegenerative Diseases/drug therapy,” “Cerebral Cortex/drug effects,” “Hippocampus/drug effects,” “Neuroprotective Agents/pharmacology,” “Disease Models, Animal,” “Alzheimer's Disease/Drug Therapy,” “Parkinson Disease/psychology,” “Brain Ischemia/drug therapy,” “Anti‐Anxiety Agents/pharmacology,” and “Antidepressive Agents/administration & dosage.” The selected articles were analyzed to evaluate the neuropharmacological activity of PNL and its derivatives. This assessment encompassed key parameters, including concentration or dose, route of administration, experimental methodologies (both in vivo and in vitro), observed pharmacological effects, and potential mechanisms of action. Additionally, studies on neurodegenerative diseases and their pathological mechanisms were reviewed to explore the therapeutic potential of PNL. The taxonomy of plant‐derived PNL compounds was validated using the World Flora Online database to ensure accurate classification, while their chemical structures were confirmed through PubChem. The most relevant data were compiled into tables and figures to facilitate comparative analysis and interpretation. This comprehensive approach enabled the identification of significant trends in the neuropharmacological properties of PNL derivatives, their potential in targeting neurodegenerative diseases, and the mechanisms underlying their therapeutic effects.

### 2.2 Inclusion Criteria

1. Studies conducted in vitro, in silico, ex vivo, or in vivo with or without laboratory animals, including mice, and rats, as well as studies involving their derived tissues or cells.
2. Research involving PNL, its derivatives, or its preparations.
3. Studies in which PNL or its derivatives exhibit joint activity with other chemical compounds.
4. Studies that propose or investigate potential mechanisms of action.

### 2.3 Exclusion Criteria

1. Studies demonstrating data duplication or those whose titles and/or abstracts did not meet the inclusion criteria.
2. Research involving PNL in contexts unrelated to neurodegenerative diseases.
3. Papers written in languages other than English.

## 3 Results and Discussion

### 3.1 Botanical Sources of Paeonol

For centuries, medicinal plants have been an important part of treatment, even before science could explain their effects [17]. Even today, nearly 80% of people worldwide still depend on herbal medicine for their health needs [18]. The demand for plant‐based treatments is growing rapidly, with the market expected to more than double from $165.66 billion in 2022 to $347.50 billion by 2029 [19]. Many modern medicines, including one in four prescription drugs, come from plant sources [20]. As antibiotic resistance and chronic diseases are rising, researchers are finding the benefits of medicinal plants to create new and effective treatments [21, 22]. PNL is a bioactive phenolic compound that is known for various pharmacological properties. It is primarily extracted from various plant sources. The most common source is *Paeonia suffruticosa* (tree peony). The root bark, dried root bark, root cortex, and cortex are mostly found to be high in PNL in multiple studies [23, 24, 25, 26, 27]. *Paeonia suffruticosa* is not only rich in PNL content, but also it has traditional uses in inflammation [28] and neuroprotection [29]. Other species of the Paeonia genus, such as *P. lactiflora, P. moutan*, and *P. ostii*, have also been reported to have PNL from their roots [30, 31, 32]. *P. lactiflora* also demonstrated anti‐inflammatory activity [33] while *P. moutan* is effective aginst neurotoxicity [34]. In addition, *Cynanchum paniculatum* (paniculate swallowwort) is another plant, which contains PNL in its root bark [35]. This plant has long been used in East Asian medicine for pain relief and wound healing [36, 37]. Various botanical sources and plant parts of PNL are provided in Table 1.

**TABLE 1:** Various botanical sources and plant parts of Paeonol.

Plant name	Plant parts	References
*Paeonia suffruticosa*	Root bark	[23]
*Paeonia suffruticosa*	Dried root bark	[24]
*Cynanchum paniculatum*	Root bark	[35]
*Paeonia lactiflora, Paeonia suffruticosa*	Root	[31]
*Paeonia ostia, P. lactiflora*	Root	[30]
*Paeonia moutan*	Root bark	[26]
*Pycnostelma paniculatum*	Herb	
*Paeonia suffruticosa*	Root	
*Paeonia suffruticosa*	Root barks	[27]
*C. paniculatum and P. suffruticosa*	—	[38]
*Paeonia lactiflora, P. albiflora, P. vitchii*	—	[39]
*P. suffruticosa*	Root cortex	[25]
*Paeonia moutan*	—	[32]
*Paeonia suffruticosa*	Cortex	[40]

### 3.2 Pharmacokinetics Study of Paeonol

A pharmacokinetic (PK) study looks at how a drug moves through the body and how it's absorbed, spread, broken down, and eliminated [41]. It's important for figuring out the best dosing, predicting how well the drug will work, and ensuring it's safe [42]. PK studies also help spot potential drug interactions, bioavailability, and half‐life, making sure the drug reaches the right place in the body at the right levels without causing harm [43]. This information is much needed for developing new drugs, getting them approved, and tailoring treatments to individual needs [44]. Our pharmacokinetic study on PNL demonstrates that it is a compound with the molecular formula C_9_H_10_O_3_ and a molecular weight of 166.17 g/mol. It contains 12 heavy atoms, including six that are part of an aromatic system. The molecule has one hydrogen bond donor and three hydrogen bond acceptors. Its molar refractivity is 45.15, and it has a moderate lipophilicity of 1.98 (Log P_o/w_). PNL also follows Lipinski's Rule of Five without any violations, indicating its drug‐like properties. Its bioavailability score is 0.55 which means it has moderate oral availability. Additionally, it is classified as soluble in water, with a predicted Log S (ESOL) value of –2.36. PNL has a Caco‐2 permeability value of 1.235 (log Papp in 10^−6^ cm/s), indicating moderate intestinal permeability. It demonstrated high human intestinal absorption at 93.774%. However, its skin permeability is relatively low, with a log K_p_ value of –2.74. Importantly, PNL does not inhibit P‐glycoprotein I or II, indicating that it is improbable to be influenced by these essential drug transporters. PNL has limited ability to cross the blood–brain barrier (log BB = 0.07) and poor permeability into the central nervous system (log PS = –2.276). The predicted volume of distribution at steady state (VDss) is –0.1 log L/kg, indicating it is not widely distributed in body tissues. PNL inhibits the CYP1A2 enzyme but does not significantly affect CYP2C19, CYP2C9, CYP2D6, or CYP3A4. Finally, the value of total clearance is 0.653 log mL/min/kg, and it is not a substrate of the renal organic cation transporter 2 (OCT2), meaning it is primarily eliminated through the kidneys. The literature review further gives us more knowledge on the pharmacokinetics of PNL. PNL was rapidly absorbed (T_max_: 7.50 ± 2.74 min) with a short elimination half‐life (t_1/2_: 59.85 ± 10.23 min) and a maximum concentration (C_max_) of 0.71 ± 0.13 mg/L. The total drug exposure (AUC_0−∞_) was 43.06 ± 6.10 mg·L/min, with a clearance rate of 0.24 ± 0.03 L/min/kg. Its absolute bioavailability after intramuscular administration was 68.68% [45]. Another pharmacokinetic study showed rapid absorption and distribution, while metabolites were mainly excreted in urine (29.8%–27.4%) and bile (8.24%–1.76%). Total metabolite recovery was around 35% within 24 h [27]. The PNL pharmacokinetic profile concludes that it has a lot of potential as a drug, with good bioavailability, and quick absorption. It shows moderate permeability in the intestines and has a low risk of interacting with other drugs, as it inhibits CYP1A2. However, it doesn't pass through the skin easily and poor at crossing the blood–brain barrier. Pharmacokinetic study of PNL by SwissADME and pkCSM is given in Table 2.

**TABLE 2:** Different pharmacokinetics parameters of Paeonol.

Properties	Factors	Paeonol
Physicochemical properties	Formula	C_9_H_10_O_3_
	Molecular weight (g/mol)	166.17
	Molar refractivity	45.15
	Number of heavy atoms	12
	Number of aromatic heavy atoms	6
	Number of H‐bond acceptors	3
	Number of H‐bond donors	1
Lipophilicity	Log Po/w (XLOGP3)	1.98
Drug‐likeness	Lipinski	Yes; 0 violation
	Bioavailability score	0.55
Water solubility	Log S (ESOL)	–2.36
	Class	Soluble
Absorption	Caco2 permeability (log Papp in 10^−6^ cm/s)	1.235
	Skin permeability (log K_p_ cm/h)	–2.74
	Intestinal absorption (human) numeric (%Absorbed)	93.774
	P‐glycoprotein II inhibitor	No
	P‐glycoprotein I inhibitor	No
Distribution	CNS permeability (log PS)	–2.276
	BBB permeability (log BB)	0.07
	VDss (human) (log L/kg)	–0.1
Metabolism	CYP3A4 inhibitor	No
	CYP2D6 inhibitor	No
	CYP2C9 inhibitor	No
	CYP2C19 inhibitor	No
	CYP1A2 inhibitor	Yes
Excretion	Renal OCT2 substrate	No
	Total clearance (log mL/min/kg)	0.653

### 3.3 Effects of Paeonol on Neurological Disorders: Underlying Mechanisms

#### 3.3.1 Age‐Related Neurodegenerative Disorders

As people get older, certain factors in the brain must work well together, like the levels of stress, enzymes, chemicals, and brain activity. If these factors are not in balance, these can lead to brain disorders like AD or PD [46]. High levels of ROS and malondialdehyde (MDA) result in oxidative stress, which is a major factor in aging and neurodegenerative diseases. Oxidative stress damages cellular components, thus causing neuronal loss and cognitive decline. SOD and GSH are antioxidants that reduce oxidative stress by converting ROS into less harmful substances [47, 48]. Another essential factor for maintaining neuronal excitability is Na^+^, K^+^‐ATPase which works by regulating ion gradients [49]. ACh is a neurotransmitter that is involved in learning and memory. AChE breaks down ACh. Thus, increased AChE activity can reduce ACh levels [50]. Recent studies investigated whether PNL has any therapeutic effect on age‐related neurodegenerative disorders. In a study involving male ICR mice (50–100 mg/kg, p.o.), PNL demonstrated improved cognitive performance by increasing antioxidants level like SOD and GSH, which are crucial for protecting the brain from oxidative damage. The increase in Na^+^, K^+^‐ATPase activity was also observed, which suggests an improvement in memory and learning, which are normally affected by aging. Additionally, PNL reduced MDA, oxidative stress, ROS, and AChE activity, indicating its effectiveness to reduce age‐related neurodegenerative symptoms. The induction of ACh and cognitive abilities further suggests that PNL could have therapeutic effects for age‐related cognitive decline by reducing oxidative damage and improving neurotransmitter function [51].

#### 3.3.2 Alzheimer's Disease (AD)

AD is a progressive neurodegenerative disorder characterized by cognitive impairment associated with extracellular amyloid‐β deposition, intracellular tau hyperphosphorylation, synaptic dysfunction, and neuronal loss [52]. The pathophysiology behind the disease is oxidative stress, inflammation in the brain, and the loss of brain cells. COX‐1 is an enzyme that plays an important role in brain inflammation and is mostly found in microglia, the brain cells that often surround the amyloid plaques seen in AD. Early signs of AD often include changes in behavior and memory, showing that thinking and learning are starting to decline [53]. In neuroapoptosis brain cells die off in a controlled way, which contributes to the loss of brain cells in AD [54]. Whereas neuroprotection refers to the brain's natural efforts to protect its cells, which might help slow down the disease [55]. Vascular actin is connected to how blood vessels in the brain respond, which can affect how amyloid plaques are cleared [56]. Changes in memory and behavior that happen in AD are described by cognitive neurobehavioral performance [57]. When microglia become activated, they contribute to the brain's inflammation and worsen the disease [58]. Neuroinflammation is long‐lasting brain inflammation that speeds up the progression of AD [59]. Recent studies suggest that PNL could be a possible treatment for AD, as it reduces inflammation, improves brain cell function, and protects against neuron damage. In an APP/PS1 double transgenic mouse model, PNL (20 mg/kg, p.o.) improved cognitive neurobehavioral performance, reduced TNF‐α, IL‐1β levels, and significantly decreased Aβ plaque burden, microglial activation, and neuronal loss [60]. Additionally, in male Sprague–Dawley rats (5 mg/kg, i.p.), PNL enhanced COX‐1, mitochondrial function, cortical cytochrome oxidase, and vascular actin expression, while reducing neuroapoptosis and offering neuroprotection, which is crucial for alleviating the neuronal loss seen in Alzheimer's disease [61]. PNL improved mitochondrial function and cortical cytochrome oxidase activity while reducing neuroapoptosis, suggesting its neuroprotective effects. These results show that PNL helps tackle both the inflammatory and cellular damage that promotes Alzheimer's disease.

#### 3.3.3 Depression and Anxiety

Depression is a mental health condition where a person feels persistently sad, empty, and loses interest in things they once enjoyed [62]. Anxiety, on the other hand, is a feeling of constant worry or dread about things that may happen in the future [63]. To help reduce depression, several factors are involved. For example, NGF can play a role in lifting mood and easing depressive symptoms [64]. Reducing excess calcium in brain cells helps prevent further stress and damage that could contribute to depression [65]. BDNF is another important factor for improving mood and reducing symptoms by supporting brain cell health [66]. For example, animals that spend more time exploring open spaces show less anxiety [67]. Anxiety is often connected to oxidative stress and inflammation in the body, so reducing these factors can help ease anxiety symptoms [68]. Research has shown that PNL can help with both depression and anxiety by reducing stress and supporting brain health. In PC12 cell lines, PNL (5–20 μM) enhanced cell viability, reduced Ca^2+^ overload and immobility time, suggesting antidepressant‐like effects. In KM mice (10–80 mg/kg in mice, p.o.) and Wistar rats (12.5–50 mg/kg in, p.o.), PNL increased NGF mRNA and improved cytoprotective effects [69]. The anxiolytic effects were further supported by a change in behavior, where Swiss mice (8.75–140 mg/kg, p.o.) spent more time in the open arms of the elevated plus maze, indicating reduced anxiety. Additionally, PNL helped reduce oxidative stress and inflammation, which is a main factor in both depression and anxiety [70]. These results show how PNL could be a promising treatment for mood disorders, helping to treat both the physical and emotional symptoms.

#### 3.3.4 Epilepsy

Epilepsy is a condition where a person experiences frequent seizures, and it is often linked to problems like oxidative stress and issues with the brain's energy‐producing parts [71]. Oxidative stress plays a critical role in seizure pathogenesis by promoting neuronal hyperexcitability and network instability. Attenuation of oxidative stress preserves neuronal integrity, stabilizes synaptic transmission, and reduces susceptibility to seizure generation [72]. Enhancement of endogenous antioxidant defenses, particularly through upregulation of SOD activity, mitigates ROS‐mediated neuronal damage. Concurrent reduction in apoptotic signaling limits neuronal loss and preserves neural circuitry integrity. Collectively, these effects contribute to anticonvulsant activity by decreasing seizure frequency and severity while preventing progressive neurodegeneration associated with recurrent seizures [73, 74, 75]. In a recent study by Liu et al. (2019) in Wistar rats (20–60 mg/kg, i.p.), PNL increased SOD and MDA levels, reduced oxidative stress, and prevented neuroapoptosis, thus helping to protect neurons from the damage caused by epileptic seizures [16]. Additionally, in a study by Ramazi et al. (2022), PNL at doses of 30–50 mg/kg (p.o.) demonstrated significant neuroprotective effects in male Wistar rats, reducing oxidative stress, pyroptosis, astrocyte activity, and inflammation. PNL also decreased seizure severity, neuronal loss, and apoptosis [76]. These results show PNL's potential as an anticonvulsant that helps to reduce oxidative damage and inflammation, which causes brain injury in epilepsy.

#### 3.3.5 Ischemic Stroke

Cerebral infarction, commonly known as an ischemic stroke, occurs when part of the brain tissue dies due to a lack of blood flow, causing brain cells to be deprived of oxygen [77]. Cerebral ischemia, the condition where there is insufficient blood flow to the brain [78]. Regulating TLR2 and TLR4 receptors, which are involved in inflammation, can help minimize the damage [79]. Blocking the NF‐κB pathway reduces neuronal harm by controlling the release of inflammatory cytokines [80, 81]. Additionally, managing the activation of microglial cells, which are part of the brain's immune response, can reduce the secondary damage [82]. Balancing IL‐1β with its natural antagonist helps reduce neuroinflammation, and decreasing the production of superoxide anions can protect the brain cells from oxidative stress [83]. Strategies like inhibiting neutrophil adhesion and managing swelling in the brain can help reduce the size of the infarction and prevent further harm [84]. PNL is observed in several studies to reduce ischemic stroke. In male SD rats (20 mg/kg, i.p.), PNL reduced cerebral infarction volume and improved neurological deficits. A decrease was also observed in microglial activation and TLR2 and TLR4 activation, which are essential in inflammatory response after stroke. Furthermore, PNL suppressed the NF‐κB signaling pathway and reduced IL‐1β levels [13]. This suggests that PNL may help reduce the extent of secondary brain injury often seen after stroke through anti‐inflammatory action. Additionally, in a study by Hsieh et al. (2006), PNL (15–20 mg/kg, i.v.) significantly reduced superoxide anion levels and microglial activation that further supports PNL efficacy for reducing neuroinflammation and infarct size [85]. Similarly, Zhao et al. (2018) observed that PNL (25 mg/kg, i.p.) in male SD rats reduced neurological impairment, infarct volume, and cerebral edema, as well as neuronal loss. Moreover, PNL reduced the activation of microglia and astrocyte proliferation, both of which contribute to the neuroinflammatory response after ischemic stroke [86]. Tang et al. (2024) revealed that a blood–brain barrier‐penetrating and lesion‐targeting nanoplatform loaded with PNL and polymetformin effectively treated ischemic stroke. PNL acted as an anti‐inflammatory and antioxidant agent, protecting neurons and reducing oxidative stress [87]. In conclusion, PNL has the potential to reduce the damage caused by ischemic stroke through its anti‐inflammatory and neuroprotective effects.

#### 3.3.6 Multiple Sclerosis

Multiple sclerosis (MS) is a chronic autoimmune disease that affects the central nervous system [88]. In MS, the immune system mistakenly attacks the protective layer around nerve fibers, disrupting communication between the brain and the rest of the body [89]. To help manage the severity of MS, reducing factors like nerve damage, inflammation, and oxidative stress is the solution. Lowering harmful substances such as MDA, ROS, and certain inflammatory markers can slow the disease's progression [90]. Additionally, increasing levels of BDNF can help support nerve health and improve resilience [91]. In a study that investigates the effect of PNL on Multiple sclerosis C57BL/6 mice were used. 25–100 mg/kg (p.o.) PNL showed a reduction in neuronal injury, lipid peroxidation, and ROS. It also reduced the levels of IL‐6, TNF‐α, and markers of depression, while also increasing BDNF and enhancing SOD activity [92]. In conclusion, the study gets down to the decision that PNL could be a promising treatment for multiple sclerosis as it reduces brain damage, inflammation, and stress while boosting neuroprotective factors.

#### 3.3.7 Mechanistic Insights Into PNL‐Mediated Neuroprotection

A complicated mix of inflammation and oxidative stress influences neurodegenerative diseases [93]. Key factors like NF‐κB, TLR4, and MyD88 promote brain inflammation by triggering certain genes that start an inflammatory process [94]. Pro‐inflammatory chemicals, including NO, iNOS, COX‐2, and PGE2, along with cytokines like IL‐1β and TNF‐α, play a role in this inflammation [95]. On the other hand, anti‐inflammatory pathways, such as AMPK‐α and blocking GSK3α/β, can help reduce this inflammation [15]. Protective enzymes like SOD and HO‐1 balance oxidative stress, caused by harmful molecules like ROS and NADPH oxidase. Strengthening these protective enzymes while lowering inflammation can help slow down neurodegenerative diseases. Additionally, adjusting certain kinases, such as p38 MAPK and STAT3, can also affect inflammation and oxidative stress [96]. PNL has demonstrated promising neuroprotective effects in the neurodegenerative diseases. An in vitro study by Lin et al. (2015) using the BV‐2 cell line has shown that PNL reduces the expression of inflammatory and oxidative stress markers such as NO, NOS, COX‐2, HO‐1, ROS, AMPK‐α, GSK 3α/β, LPS/INF‐γ, p38, and STAT3 [15]. Similarly, in another study by He et al. (2016) in the N9 cell line, PNL decreased levels of NO, PGE2, COX‐2, iNOS, IL‐1β, TNF‐α, NF‐κB, TLR4, MyD88, IRAK4, TRAF6, p‐IkB‐α, NF‐kB p65, p‐P38, p‐JNK, and p‐ERK [97]. Moreover, an in vivo study involving pregnant Sprague–Dawley rats demonstrated that PNL increased antioxidant enzymes like SOD and Bcl‐2 while reducing oxidative stress and inflammation markers such as ROS, COX‐2, iNOS/NO, NADPH, PGE2, MAPK, and NOX2 [98]. These findings indicate that PNL could be an essential therapeutic agent for managing neurodegenerative diseases.

#### 3.3.8 Neuropathic Pain

Neuropathic pain is a chronic condition that results from damage or disease to the somatosensory nervous system [99]. It is often caused by factors like physical injury, diabetes, herpes zoster, or cancer treatments [100]. An increase in IL10, CD206, ARG‐1, TGF‐β, and M2 microglia promotes anti‐inflammatory responses and neuroprotection, thus reducing pain [101]. Conversely, decreasing pro‐inflammatory markers like IL1β, iNOS, CD32, IL6, IBA‐1, RhoA, RhoA‐GTP, COX2, Rock1, and p‐p38MAPK, along with the modulation of M1 microglia, can reduce inflammation and pain signaling, contributing to pain relief [102]. An investigation on male SD rats, PNL (1–20 μM, i.p.) modulated microglial polarization by making a shift from M1 to M2 microglia. By increasing M2 markers like IL‐10, CD206, and TGF‐β, while decreasing M1 markers like IL‐1β and COX‐2, PNL reduced neuroinflammation and neuropathic pain [103]. In conclusion, PNL is effective in treating neuropathic pain.

#### 3.3.9 Neurotoxicity

Neurotoxicity refers to the damage caused to the brain and nervous system by harmful substances, whether they are biological, chemical, or physical [104]. These harmful agents, known as neurotoxins, can lead to the death or dysfunction of neurons, which are necessary for transmitting signals in the nervous system [105]. Antiapoptotic proteins like Bcl‐2 and Bcl‐xL act like bodyguards for our cells. They prevent neurons from dying under stress and promotes the survival [106]. Additionally, molecules like phosphorylated ERK1/2 (pERK1/2) can activate other protective pathways, boosting the cell's ability to resist damage [107]. Histone acetylation, involving Ac‐H3H9 and Ac‐H4K12, helps by turning on genes that protect neurons, allowing them to function properly [108]. Finally, inhibiting HDAC2 and HDAC3, enzymes that regulate gene expression, can help keep neurons safe by promoting better cell function and preventing neurodegeneration [109]. In pregnant Sprague–Dawley rats (50–150 mg/kg, p.o.), PNL reduced neuroapoptosis by increasing levels of anti‐apoptotic proteins and reducing pro‐apoptotic factors like Bad and Bax. It also increased phospho‐ERK1/2, Ac‐H3H9, and Ac‐H4K12 (Jin et al., 2020). Zhang et al. (2016) also observed a reduction in neuroapoptosis in pregnant Sprague–Dawley rats where PNL (20–80 mg/kg) increased anti‐apoptotic proteins like Bcl‐xL and Bcl‐2 while reducing pro‐apoptotic markers such as Bax, Bad, p‐JNK, p‐p38, and caspase‐3 [110]. These findings propose PNL's neuroprotective efficacy in reducing neurotoxic damage.

#### 3.3.10 Parkinson's Disease (PD)

PD is a progressive condition that affects the brain, leading to the gradual loss of dopamine‐producing neurons in the substantia nigra [111]. This results in symptoms like tremors, stiffness, slow movements, and trouble with balance [112]. Several factors can help slow the progression of PD. Reducing oxidative stress, which causes damage to neurons through harmful molecules like ROS, can help protect the brain cells [113]. Lowering neuroinflammation, which is when the brain's immune cells become overactive, can slow the disease down by reducing the damage they cause [114]. Boosting neurotrophic factors, which are proteins that help neurons survive and grow, can also protect these cells [115]. Increasing the number of TH‐positive neurons, which are important for making dopamine, can help with motor symptoms [116]. Higher levels of the antioxidant enzyme SOD can protect the brain by reducing oxidative stress [117]. Reducing motor complications, like involuntary movements or fluctuations in motor control from long‐term treatment, can make life easier for people with PD [118]. Inhibiting apoptosis, or programmed cell death, also helps protect neurons from degeneration. Reducing pro‐inflammatory molecules like IL‐1β and iNOS can reduce inflammation and protect the brain from further damage [119]. Literature studies on PNL indicate that it is effective in treating PD. In a study by Shi et al. (2016) on C57BL/6 mice treated with PNL (20 mg/kg, p.o.), the compound reduced oxidative stress and neuroinflammation while increasing neurotrophic effects [120]. Similarly, in another study on adult Wistar rats (100 mg/kg, p.o.), PNL enhanced the number of TH‐positive neurons and reduced motor complications, DNA fragmentation, and markers of apoptosis, including IL‐1β and iNOS [121]. These results demonstrate PNL's potential to improve motor function and protect the brain in PD.

#### 3.3.11 Post‐Stroke Dementia (PSD)

PSD is a type of dementia that develops after a stroke, and it can include conditions like vascular dementia and Alzheimer's disease [122]. Bcl‐2 can help protect neurons from damage after a stroke, potentially preserving brain function and lowering the chances of dementia [123]. SOD reduces oxidative stress in the brain, and higher levels of SOD have been linked to better cognitive recovery after a stroke, offering protection against cognitive decline [124]. Reducing apoptosis is also crucial, as it helps preserve brain tissue and function, making dementia less likely [125]. Additionally, managing the production of amyloid beta (Aβ), a protein associated with Alzheimer's, might help prevent cognitive decline by reducing its buildup in the brain [126]. Finally, targeting factors like apoptosis‐inducing factor (AIF) could further help preserve neurons, making it easier to protect against poststroke dementia [127]. An investigation on male SD rats treated with PNL (20 mg/kg, i.p.) demonstrated PNL increased Bcl‐2 and SOD levels and reduced Bax, APP, BACE, and Aβ levels [14]. These findings indicate that PNL reduced apoptosis and cognitive impairments. Thus, PNL is effective in PSD treatment.

#### 3.3.12 Traumatic Brain Injury (TBI)

TBI happens when an external force, like a blow to the head from an accident or fall, causes damage to the brain [128]. It can lead to both short‐term and long‐term problems with thinking, movement, and emotions [129]. Neuroprotection strategies, such as using treatments like BDNF and NGF, help protect brain cells and promote healing [130]. Inflammation, which occurs when brain cells called microglia become activated and release chemicals, can worsen the damage, so reducing this inflammation is important [131]. Swelling, or edema, is another common issue after TBI and can put pressure on the brain, so managing it early is key to preventing further harm [132]. Microglial activation can help clear up debris and promote repair [133]. In Wistar rats (50 mg/kg, p.o.), PNL reduced inflammation, microglial activation, and cerebral cortex damage. Thus, it shows neuroprotective effects [134]. This suggests that PNL can reduce the secondary injury after TBI and help in faster recovery and reducing long‐term damage.

#### 3.3.13 Neuroimmune Regulation

Neuroimmune regulation is the complex bidirectional communication between the nervous and immune systems, which maintains homeostasis and coordinates responses to stress, injury, and infection [135]. This interaction involves signaling molecules such as cytokines, neurotransmitters, and neuropeptides that influence both neural activity and immune function [135]. Proper neuroimmune regulation is essential for maintaining brain health, preventing neuroinflammation, and protecting against neurodegenerative and psychiatric disorders, as dysregulation can lead to chronic inflammation, neuronal damage, and the progression of diseases such as Alzheimer's, Parkinson's, and multiple sclerosis [136]. Interestingly, natural products have demonstrated the ability to modulate neuroimmune pathways, restoring immune balance and providing significant neuroprotective effects [137]. PNL demonstrates a strong immunomodulatory effect by suppressing pro‐inflammatory mediators (IL‐1β, TNF‐α, and IL‐6) and enhancing anti‐inflammatory cytokines (IL‐10 and TGF‐β), thereby restoring immune balance within the central nervous system [92, 110]. It modulates the TLR4/MyD88/NF‐κB signaling pathway, limiting microglial overactivation and increasing neuroimmune regulatory potential [13, 97]. These emerging findings highlight PNL's systemic neuroimmune regulatory potential, representing a frontier mechanism that may explain its broad neuroprotective efficacy.

**FIGURE 1:**
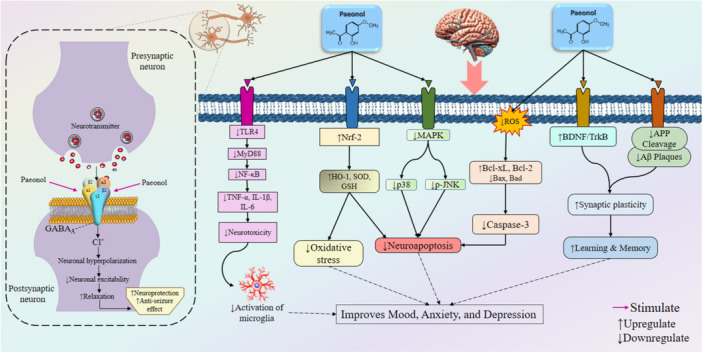
Proposed mechanisms underlying the neuroprotective effects of PNL. [PNL exerts multi‐target activity by inhibiting oxidative stress and neuroinflammation while preventing neuronal apoptosis. It modulates key pathways such as NF‐κB, MAPK, TLR2/4, and AMPK, leading to reduced expression of iNOS, COX‐2, and pro‐inflammatory cytokines (TNF‐α and IL‐1β). Paeonol enhances the activity of antioxidant enzymes (SOD and GSH) and upregulates anti‐apoptotic proteins (Bcl‐2 and HO‐1) while suppressing pro‐apoptotic markers (Bax and Caspase‐3). NF‐κB, nuclear factor kappa‐light‐chain‐enhancer of activated B cells; MAPK, mitogen‐activated protein kinase; TLR, toll‐like receptor; AMPK, AMP‐activated protein kinase; iNOS, inducible nitric oxide synthase; COX‐2, cyclooxygenase‐2; TNF‐α, tumor necrosis factor‐alpha; IL‐1β, interleukin‐1 beta; SOD, superoxide dismutase; GSH, glutathione; Bcl‐2, B‐cell lymphoma 2; HO‐1, heme oxygenase 1; Bax, Bcl‐2‐associated X protein].

PNL exhibits broad neuroprotective potential across various neurological disorders by reducing oxidative stress, inflammation, and neuronal apoptosis. It enhances antioxidant defenses (SOD and GSH), improves mitochondrial and neurotransmitter function, and regulates neuroimmune responses through pathways like TLR4/MyD88/NF‐κB. PNL has shown therapeutic effects in Alzheimer's, Parkinson's, epilepsy, stroke, multiple sclerosis, and mood disorders by restoring cellular balance and promoting neuronal survival. It modulates microglial activation, enhances neurotrophic factors (BDNF and NGF), and inhibits pro‐inflammatory cytokines (IL‐1β, TNF‐α, and IL‐6). Overall, PNL's antioxidant, anti‐inflammatory, and immunomodulatory actions make it a promising natural agent for preventing and treating neurodegenerative and neuropsychiatric diseases. However, the detailed neurological activity of PNL is summarized in Table 3, while overall neuroprotective mechanisms of PNL, including its antioxidant, anti‐inflammatory, and anti‐apoptotic pathways, are illustrated in Figure 1. The neuroprotective effects of PNL across various neurological disorders are comparatively summarized in Table 4.

**TABLE 3:** Neurological activity and mechanism of Paeonol.

Disease name	Experimental model/cell lines	Tested concentrations, (R/A)	IC_50_	Results/mechanisms	References
Age‐related neurodegenerative disorders	Male ICR mice, in vivo (*n* = 10–12)	50–100 mg/kg (p.o.)	—	↑ SOD, GSH, Na+, K+ ‐ATPase activities, acetylcholine (Ach), learning and memory ability	[51]
				↓MDA, Oxidative stress, ROS, AChE activity	
Alzheimer's disease	APP/PS1 (APPswe, PSEN1dE9) double transgenic mice, in vivo (*n* = 18)	20 mg/kg (p.o.)	—	↑Cognitive neurobehavioral performance	[60]
				↓TNF‐α, IL‐1β, Aβ plaque burden, microglia activation, neural loss, neuroinflammation	
Alzheimer's disease	Male Sprague–Dawley rats, in vivo (*n* = 8)	5 mg/kg (i.p.)	—	↑COX‐1, Mitochondrial Dysfunction, Behavior and learning, neuroprotection, cortical cytochrome oxidase, vascular actin	[61]
				↓Neuroapoptosis	
Depression	Male SD (Sprague–Dawley) rats, in vivo	25–80 mg/kg (i.g.)	—	↑BDNF, Rac1	[138]
				↓RhoA, Cofilin1 Activity, Depressive‐like behaviors, dendritic atrophy, dendritic spine loss	
Depression	PC12 cells line, in vitro	5–20 μM	—	↑NGF mRNA, cytoprotective effects, cell viability	[69]
	Male KM (Kunming Mice) mice and male Wistar rats, in vivo (*n* = 10)	10–80 mg/kg in mice (p.o.) and 12.5–50 mg/kg in rats (p.o.)			
				↓Ca^2+^ overload, LDH, Immobility time, depression, cytotoxicity	
Anxiolytic	Male Swiss mice, in vivo (*n* = 10)	8.75–140 mg/kg (p.o.)	—	↑Percentage of time spent in open arms in the elevated plus maze, the time spent in the light area	[70]
				↓Anxiety, oxidative stress, inflammation	
Epilepsy	Wistar rats, in vivo (*n* = 6)	20–60 mg/kg (i.p.)	—	↑SOD, MDA	[16]
				↓Oxidative stress, apoptosis,	
Neuroprotective and anticonvulsant effects	Male Wistar rats, in vivo	30 and 50 mg/kg	—	↑Neuro‐protection,	[76]
				↓Oxidative stress, pyroptosis, astrocyte activity, apoptosis, seizure severity, neuronal loss, inflammation	
Ischemic stroke	Male SD (Sprague–Dawley) rats, in vivo (*n* = 12)	20 mg/kg (i.p.)	—	↓Cerebral infarction volume, neurological deficits, TLR2, TLR4, NF‐*κ*B, microglial activation, IL‐1*β*	[13]
Cerebral infarction	Male SD rats, in vivo (*n* = 6)	15–20 mg/kg (i.v.)	—	↓Superoxide anion, IL‐1β,	[85]
				microglia activation, Cerebral infarction, neuro‐deficit	
Cerebral ischemia	Male SD (Sprague–Dawley) rats, in vivo (*n* = 8–26)	25 mg/kg (i.p.)	—	↓Neurological impairment, Infarct volume, Cerebral edema, Neuronal loss, microglial activation and astrocyte proliferation	[86]
Multiple sclerosis	Male C57BL/6 mice, in vivo (*n* = 13)	25 and 100 mg/kg (p.o.)	—	↓ Neuronal injury, MDA, lipid peroxidation, ROS, IL‐6 and TNF‐α level, depression, immobility duration, LPS, inflammation	[92]
				↑BDNF level, Superoxide Dismutase	
Neurodegenerative disease	BV‐2 cell line, in vitro	3–30 μM	—	↓NO, NOS, COX‐2, HO‐1, ROS, AMPK‐α, GSK 3α/β, LPS/INF‐γ, p38, STAT3	[15]
	Male ICR (imprinting control region), in vivo	3–30 μM (i.p.)			
Neurodegenerative disease	N9 cell line, in vitro	0.12–75 μM	—	↓NO, PGE2, COX‐2, iNOS, IL‐1β, TNF‐α, NF‐κB, TLR4, MyD88, IRAK4, TRAF6, p‐IkB‐α, NF‐kB, p65, p‐P38, p‐JNK, p‐ERK	[97]
Neurodegenerative disorders	Pregnant SD (Sprague–Dawley) rats, in vivo	0.75–1.5 μM (p.o.)	—	↑SOD, Bcl‐2, HO‐1	[98]
				↓ROS, COX‐2, iNOS/NO, NADPH, PGE2, MAPK, NOX2, oxidative stress, cytotoxicity	
Neuropathic pain	Male SD (Sprague–Dawley) rats, in vivo (*n* = 8),	1–20 μM (i.p.)	—	↑IL10, CD206, ARG‐1, TGF‐β, M2 microglia	[103]
				↓IL1β, iNOS, CD32, IL6, IBA‐1, RhoA, RhoA‐GTP, COX2, Rock1, and p‐p38MAPK, M1 microglia	
Neurotoxicity	Pregnant Sprague–Dawley rats, in vivo (*n* = 6)	50–150 mg/kg (p.o.)	—	↑Bcl‐2, Bcl‐xL, IAPs‐ xIAP, c‐IAP‐1 and c‐IAP‐2, survivin, phospho‐ERK1/2, Ac‐H3H9 and Ac‐H4K12	[108]
				↓Bad, Bax, neuroapoptosis, JNK and p38MAPK, HDAC2 and HDAC3	
Neuroapoptosis and cognitive dysfunctions	Pregnant Sprague–Dawley rats, in vivo (*n* = 6)	20–80 mg/kg	—	↑Bcl‐xL, Bcl‐2, learning and memory	[110]
				↓Bax, Bad., p‐JNK, p‐p38, caspase‐3, NF‐κB, p65, p‐Iκ‐Bα, TNF‐α, IL‐1 β and IL‐6, Cytochrome c, Neuroapoptosis	
Parkinson's disease	Male C57BL/6 mice, in vivo (*n* = 10)	20 mg/kg (p.o.)	—	↓Oxidative stress, neuroinflammation	[120]
				↑ neurotrophic effect	
Parkinson's disease	Adult rats (a total of 72 male Wistar strain, in vivo (*n* = 8)	100 mg/kg (p.o.)	—	↑ TH‐positive neurons, SOD	[121]
				↓GFAP, Motor complications, DNA fragmentation, apoptosis, IL‐1β, iNOS, pASK1	
Poststroke dementia	male SD (Sprague–Dawley) rats, in vivo (*n* = 6)	20 mg/kg (i.p.)	—	↑Bcl‐2, SOD	[14]
				↓Apoptosis, Bax, APP, BACE, AIF, Aβ	
Traumatic brain injury	Male Wistar albino rats, in vivo (*n* = 8)	50 mg/kg p.o.	—	↑Neuroprotection, ↓Inflammation, edematous effects, microglial activation, Cerebral cortex damage	[134]

Abbreviations: ↑, increase/stimulation/upregulation/initiation; ↓, decrease/inhibition/downregulation/blocking; Ac‐H3H9, acetylation of histone H3 at lysine 9; Ac‐H4K12, acetylation of histone H4 at lysine 12; Ach, acetylcholine; Aβ, amyloid‐beta; AIF, apoptosis‐inducing factor; AMPK‐α, AMP‐activated protein kinase alpha; APP, amyloid precursor protein; ARG‐1, arginase‐1; BACE, beta‐secretase; Bax, Bcl‐2‐associated X protein; Bcl‐2, B‐cell lymphoma 2; BDNF, brain‐derived neurotrophic factor; c‐IAP‐1, cellular inhibitor of apoptosis protein 1; c‐IAP‐2, cellular inhibitor of apoptosis protein 2; CD206, cluster of differentiation 206; CD32, cluster of differentiation 32; COX‐1, cyclooxygenase‐1; COX‐2, cyclooxygenase‐2; GFAP, glial fibrillary acidic protein; GSH, glutathione; HO‐1, heme oxygenase‐1; IBA‐1, ionized calcium binding adapter molecule 1; iNOS, inducible nitric oxide synthase; IL‐1β, interleukin‐1 beta; INF‐γ, interferon‐gamma; LDH, lactate dehydrogenase; M2 microglia, M2 macrophage‐like microglia; MDA, malondialdehyde; Na+, K+ ‐ATPase, sodium‐potassium ATPase; NF‐κB, nuclear factor kappa‐light‐chain‐enhancer of activated B cells; NGF, nerve growth factor; NO, nitric oxide; NOS, nitric oxide synthase; p‐ERK, phosphorylated extracellular signal‐regulated kinase; p‐Iκ‐Bα, phosphorylated inhibitor of kappa B alpha; p‐JNK, phosphorylated c‐Jun N‐terminal kinase; p‐p38, phosphorylated p38 mitogen‐activated protein kinase; p38, p38 mitogen‐activated protein kinase; Rac1, Ras‐related C3 botulinum toxin substrate 1; RhoA, Ras homolog family member A; RhoA‐GTP, RhoA‐guanosine triphosphate; ROS, reactive oxygen species; SOD, superoxide dismutase; STAT3, signal transducer and activator of transcription 3; Survivin, baculoviral IAP repeat containing 5; TGF‐β, transforming growth factor beta; TLR2, toll‐like receptor 2; TLR4, toll‐like receptor 4; xIAP, X‐linked inhibitor of apoptosis protein.

**TABLE 4:** Comparative summary of Paeonol's neuroprotective effects across neurological disorders.

Neurological disorder	Key neuroprotective effects	Primary mechanisms	Therapeutic outcomes	References
Alzheimer's disease (AD)	↓Aβ plaques, neuroinflammation, neuronal loss	↓NF‐κB, TLR4/MAPK pathways, TNF‐α, IL‐1β	Improved cognitive neurobehavioral performance; reduced microglial activation and amyloid burden	[60, 61]
	↑Cognitive function			
		↑COX‐1, mitochondrial function		
Parkinson's disease (PD)	↓Oxidative stress, motor complications	↓IL‐1β, iNOS, GFAP, DNA fragmentation, apoptosis	Protection of dopaminergic neurons; improved motor function; reduced neuroinflammation	[120, 121]
	↑TH‐positive neurons			
		↑SOD, neurotrophic factors		
Ischemic stroke	↓Infarct volume, cerebral edema, neurological deficits	↓TLR2/4, NF‐κB signaling, microglial activation, superoxide anion, IL‐1β	Reduced secondary brain injury; improved neurological recovery; decreased infarction size	[13, 85, 86]
Epilepsy	↓Seizure severity, neuronal loss, apoptosis	↓Oxidative stress, pyroptosis, astrocyte activity	Anticonvulsant effects; neuroprotection against seizure‐induced damage	[16, 76]
		↑ SOD, MDA		
Depression	↓Depressive behaviors	↓RhoA, Cofilin1 activity, dendritic atrophy and spine loss, Ca^2+^ overload	Antidepressant‐like effects; improved cytoprotective outcomes	[69, 138]
	↑BDNF, NGF, mRNA			
Anxiety	↑ Time in open arms (EPM), light area exploration	↓Oxidative stress, inflammation	Anxiolytic effects; reduced anxiety‐like behaviors	[70]
Multiple sclerosis (MS)	↓Neuronal injury, demyelination, cognitive deficits,	↓MDA, ROS, IL‐6, TNF‐α, Bax, APP, BACE, AIF, Aβ, lipid peroxidation	Reduced brain damage and inflammation; improved neuroprotection	[92]
		↑BDNF, SOD		
		activity		
Post‐stroke dementia (PSD)	↓Cognitive impairment, apoptosis	↑Bcl‐2, SOD;	Preservation of cognitive function; reduced amyloid pathology	[14]
Traumatic brain injury (TBI)	↓Inflammation, cerebral cortex damage	↓Microglial activation, edematous effects	Neuroprotection; reduced secondary injury	[134]
Neuropathic pain	↓Pain behaviors, neuroinflammation	↓M1 markers (IL‐1β, COX‐2), RhoA/p38MAPK	Pain relief through microglial polarization shift	[103]
		↑M2 microglia markers (IL‐10, CD206, TGF‐β)		
Age‐related disorders	↑Cognitive performance, memory and learning	↓MDA, ROS, AChE activity	Enhanced antioxidant defense; improved neurotransmitter function	[51]
		↑SOD, GSH, Na^+^, K^+^‐ATPase		

Abbreviations: ↑, increase/stimulation/upregulation; ↓, decrease/inhibition/downregulation; Aβ, amyloid‐beta; AChE, acetylcholinesterase; AIF, apoptosis‐inducing factor; APP, amyloid precursor protein; BACE, beta‐secretase; Bax, Bcl‐2‐associated X protein; Bcl‐2, B‐cell lymphoma 2; BDNF, brain‐derived neurotrophic factor; COX‐1, cyclooxygenase‐1; COX‐2, cyclooxygenase‐2; CD206, cluster of differentiation 206; CD32, cluster of differentiation 32; EPM, elevated plus maze; GFAP, glial fibrillary acidic protein; GSH, glutathione; HO‐1, heme oxygenase‐1; IL‐1β, interleukin‐1 beta; IL‐6, interleukin‐6; IL‐10, interleukin‐10; iNOS, inducible nitric oxide synthase; MAPK, mitogen‐activated protein kinase; MDA, malondialdehyde; NF‐κB, nuclear factor kappa‐light‐chain‐enhancer of activated B cells; NGF, nerve growth factor; ROS, reactive oxygen species; SOD, superoxide dismutase; TGF‐β, transforming growth factor beta; TH, tyrosine hydroxylase; TLR2/4, toll‐like receptor 2/4; TNF‐α, tumor necrosis factor‐alpha; TBI, traumatic brain injury; MS, multiple sclerosis; PSD, post‐stroke dementia.

### 3.4 Pharmacological Relevance

PNL is a natural substance found in plant and is known for its many health benefits. It has diverse pharmacological properties. Scientists have studied it for its ability to reduce inflammation, protect the brain, support heart health, and even fight cancer. PNL, derived from *Paeonia suffruticosa*, reduces osteoarthritis inflammation by inhibiting NF‐κB and PI3K/Akt pathways. It was administered at 30 mg/kg daily in mice osteoarthritis models [139]. PNL showed its cardioprotective effects by affecting the electrical activity of heart muscle cells by blocking sodium channels. It was tested at concentrations of 160 and 640 µM [140]. In another study, the antiangiogenesis effect of PNL was tested on HUVECs and HT1080 human fibrosarcoma cells, where it suppresses the formation of new blood vessels by inhibiting cell growth and movement. It works by inhibiting the Akt signaling pathway and reducing the expression of matrix metalloproteinases [141]. Additionally, PNL protects skin from UVB‐induced aging by inhibiting MAPK and AP‐1 pathways and activating the Nrf2 antioxidant pathway. It was tested in keratinocytes and hairless mice [142]. PNL inhibits gastric cancer cell growth by reducing proliferation and inducing apoptosis. PNL was tested in MFC and SGC‐7901 cells. In vivo, it was administered to MFC tumor‐bearing mice, significantly reducing tumor growth [143]. In a study by Ou et al. (2014), PNL was tested in EMT6 breast cancer‐bearing mice at dosages of 150 and 300 mg/kg, significantly reducing tumor weight. PNL inhibits breast cancer growth by inducing apoptosis [144]. Furthermore, PNL is effective against various more cancers, including colon [145], hepatocellular carcinoma [146], prostate cancer [147], esophageal cancer [148], and ovarian cancer [149]. PNL inhibits osteoclastogenesis and bone resorption by suppressing RANKL‐induced ERK, p38, and NF‐κB signaling [150]. In high glucose‐treated glomerular mesangial cells (GMCs) and streptozotocin‐induced diabetic mice, PNL delays diabetic nephropathy by inhibiting fibrosis and activating the Nrf2/ARE pathway [151]. Another research study conducted by Zhao et al. (2018) in a middle cerebral artery occlusion (MCAO) model using male Sprague–Dawley rats, PNL (25 mg/kg) protects against subacute and chronic ischemic stroke by reducing neuronal damage, microglial activation, and astrocyte proliferation [86]. In an STZ‐induced diabetic rat model of encephalopathy, PNL (50 and 100 mg/kg) improves pathological damage by modulating the AGEs/RAGE/NF‐κB pathway [152]. Finally, in a CUMS rat model of depression, PNL improves behavioral and neuronal damage by regulating the BDNF‐Rac1/RhoA signaling pathway [138]. PNL works by influencing several biological pathways, such as NF‐κB, PI3K/Akt, MAPK, Nrf2/ARE, and BDNF‐Rac1/RhoA. These pathways play an important role in regulating inflammation, cell survival, and stress responses. Because PNL has so many beneficial effects on the body, including reducing inflammation, protecting the brain, and fighting cancer, it can be used in medicine, but more research and clinical trials are necessary to ensure its safety.

### 3.5 Toxicological Profile

Toxicological analysis is essential in drug development to assess the safety of new treatments, whether synthetic or natural, before patient use [153]. Toxicity testing is important to ensure the safety of chemicals [154]. Moreover, it helps to adjust dosages for effective treatment and observe if there are any possible side effects early on [155]. PNL's safety was evaluated through computer simulations, single high‐dose exposure, and long‐term daily dosing in rats. In silico toxicity predictions indicated that PNL is non‐toxic, showing no risk of liver damage, cancer, immune toxicity, or genetic mutations. The estimated maximum tolerated human dose was 0.813 log mg/kg/day, with low environmental toxicity (Tetrahymena pyriformis: 0.511 log μg/mL; minnow toxicity: 0.188 log/mM). In the acute toxicity study, female Sprague–Dawley rats (*n* = 3 per group) were given single doses of 300, 2000, or 5000 mg/kg and monitored for 14 days. No toxicity signs, weight loss, or behavioral changes were observed, and all animals survived. Necropsy revealed no organ abnormalities, confirming 5000 mg/kg as the maximum tolerated dose (MTD). For long‐term safety, male and female rats (*n* = 40, 10 per group) received 50, 100, or 200 mg/kg/day for 28 days. They showed no changes in body weight, food/water intake, or behavior. Blood tests were normal, with stable WBC (16.36–19.76 × 10^3^/μL), RBC (7.20–8.18 × 10^6^/μL), hemoglobin (13.26–15.62 g/dL), ALT (45.59–65.18 IU/L), AST (87.69–140.76 IU/L), and kidney function markers (BUN: 14.89–20.18 mg/dL, creatinine: 0.484–0.654 mg/dL). Organ weights were unchanged, and no damage or inflammation in major organs was observed in microscopic examination [156]. In another hepatotoxicity model, Morsy et al. (2022) demonstrated that oral administration of PNL (100 mg/kg for 10 days) helped protect against methotrexate‐induced liver damage. Additionally, PNL did not cause any intrinsic liver toxicity [157]. In a similar study, Al‐Taher et al. (2020) showed that PNL showed cardioprotection at a dose of 100 mg/kg without any toxicity [158]. Nam et al. (2013) conducted neuroprotection studies that revealed dose‐dependent effects of PNL. At doses between 0.5 and 1 mM, PNL suppressed LPS‐induced neuroinflammation in microglia and protected hippocampal neurons, though cytotoxicity was observed at higher doses (≥ 2.5 mM) [159]. Furthermore, a study demonstrated that intraperitoneal administration of PNL (20 mg/kg for 3 days) decreased cisplatin‐induced kidney damage by reducing creatinine/BUN levels and improving survival rates (28% vs. 0% with 30 mg/kg cisplatin), all without causing hepatorenal toxicity [160]. Finally, acute toxicity testing of PNL showed LD_50_ values of 490 mg/kg (p.o.), 781 mg/kg (i.p.), and 196 mg/kg (i.v.) (https://pubchem.ncbi.nlm.nih.gov/compound/11092#section=Toxicity, accessed on March 25, 2025). The data show that PNL is generally safe, as multiple studies confirm no major toxicity. In animal tests, there were no signs of harm, and it did not affect organ function or behavior. However, in neuroprotection studies, higher doses (≥ 2.5 mM) did show some toxicity. Thus, it is important to find the right dose to get maximum benefits without any harm.

### 3.6 Clinical Evidence

Although PNL has shown remarkable pharmacological potential in preclinical and experimental models, clinical evidence supporting its efficacy in humans remains limited. A phase 2a clinical trial investigated APPA (a combination of apocynin and PNL) in 152 patients with knee osteoarthritis (Kellgren–Lawrence grades 2–3) and moderate pain, where participants received 800 mg twice daily for 28 days. The study found no significant overall improvement in WOMAC pain, function, or total scores (mean difference −0.89, 95% CI: −5.62, 3.84, *p* = 0.71), but subgroup analysis revealed meaningful benefits among patients with nociplastic or neuropathic pain. The treatment was generally well tolerated, showing only mild gastrointestinal side effects [161]. Another clinical study examined the PNL Dripping Pill (CPDP) for unstable angina and reported a 93.3% effectiveness rate in symptom improvement, along with significant reductions in plasma total cholesterol and inflammatory markers compared to conventional therapy and Tongxinluo [162]. These findings suggest that while PNL exhibits promising therapeutic potential in cardiovascular and pain‐related disorders, its clinical outcomes are inconsistent. Hence, further large‐scale, controlled, and disease‐specific clinical trials are required to better understand its mechanisms, optimize its formulations, and establish PNL as a reliable adjunct or combination therapy in human diseases.

Although substantial evidence supports the neuroprotective efficacy of PNL in experimental models, clinical translation remains limited. Recent clinical investigations involving paeonol‐containing formulations have demonstrated favorable safety profiles and therapeutic benefits in conditions such as unstable angina and osteoarthritis. Nevertheless, clinical studies directly evaluating paeonol in neurological disorders are still lacking. This highlights a critical translational gap between preclinical efficacy and clinical application. Future research should prioritize well‐designed, multicenter randomized controlled trials to evaluate the efficacy, safety, pharmacokinetics, and optimal dosing of paeonol in patients with neurodegenerative and neuropsychiatric disorders. Additionally, advanced drug‐delivery strategies aimed at improving blood–brain barrier penetration and systemic bioavailability may further facilitate the clinical development of PNL‐based therapeutics.

### 3.7 The Dose‐Effect Relationship of Paeonol

The dose‐response effects of PNL have been consistently observed across various in vitro and in vivo neurological models. In cell line studies, PNL at low micromolar concentrations (0.12–75 μM) effectively reduced inflammatory mediators such as NO, iNOS, COX‐2, PGE2, and proinflammatory cytokines (IL‐1β and TNF‐α), while enhancing antioxidant and cytoprotective factors like SOD, Bcl‐2, and NGF mRNA [76, 97]. In rodent models, moderate to higher doses (ranging from 0.75 μM to 100 mg/kg, administered orally or intraperitoneally) demonstrated significant neuroprotective effects. These effects included reduction of oxidative stress, apoptosis, neuroinflammation, microglial activation, and infarct volume, alongside improvements in antioxidant defenses (SOD and GSH), neurotrophic factors (BDNF), and behavioral or cognitive functions [61, 120, 134]. Specifically, doses of 20–60 mg/kg were effective in epilepsy and ischemic stroke models [16], 20–100 mg/kg improved outcomes in Parkinson's disease [121], depression, and multiple sclerosis models, and 50–100 mg/kg supported recovery in age‐related neurodegenerative disorders [92, 138]. Overall, PNL exhibits a clear dose‐dependent relationship, where increasing concentrations or doses correlate with enhanced neuroprotective, antioxidant, and anti‐inflammatory effects across diverse neurological disorders.

### 3.8 Overall Study, Gaps in Literature, and Future Research Directions

PNL exhibits strong preclinical evidence of neuroprotection through antioxidant, anti‐inflammatory, and anti‐apoptotic mechanisms, yet its clinical translation remains limited. The findings summarized in this review collectively indicate that PNL targets multiple signaling pathways, including NF‐κB, MAPK, TLR4, and BDNF‐related cascades, which are known to play major roles in neurodegenerative and neuropsychiatric disorders. These molecular mechanisms align closely with current therapeutic goals in diseases such as Alzheimer's, Parkinson's, and post‐stroke dementia, suggesting that PNL could serve as a valuable adjunct or lead compound for multi‐target therapy. Clinically, PNL's potential lies in its ability to modulate neuroinflammation and oxidative stress, two major drivers of disease progression in neurological disorders. However, its low blood–brain barrier permeability and moderate bioavailability limit its direct translation. Future formulations employing nanocarriers, liposomes, or solid lipid nanoparticles could enhance its brain targeting efficiency. Furthermore, combining PNL with agents such as curcumin, resveratrol, or quercetin may produce synergistic effects and broaden its therapeutic applications. Emerging evidence also supports PNL's relevance beyond neurodegeneration, including its cardioprotective, anticancer, and anti‐diabetic properties, which further justify its consideration for comorbid conditions often present in elderly patients. Clinical findings, although limited, suggest good safety and tolerability, particularly in cardiovascular disorders and osteoarthritis, indicating its potential for repurposing in neurological indications. To achieve clinical validation, large‐scale, controlled human trials and pharmacokinetic optimization studies are required.

Previous reviews on PNL have mainly concentrated on its general pharmacological activities or on specific disorders such as Alzheimer's or Parkinson's disease [163]. However, none have combined pharmacokinetic, pharmacological relevance, botanical source, toxicological properties, and clinical evidence to explain how paeonol exerts multi‐target neuroprotective actions across diverse neurological diseases. While preclinical studies on PNL have shown beneficial result, several challenges reduce its transition to clinical applications. Most of the current evidence comes from animal and laboratory studies, with few human trials and inconsistent findings, especially in neuropsychiatric disorders. One major issue is PNL's low ability to cross the blood–brain barrier though it has effective neuroprotective benefits. Thus, there is a need for improved drug delivery methods for PNL. Additionally, literature focuses on isolated molecular pathways without considering the broader biological interactions or long‐term effects. Key aspects such as optimal dosage, gender differences, and potential interactions with other drugs are not explored yet. To bridge these gaps, future studies should prioritize large‐scale clinical trials targeting specific neurological conditions. Moreover, it should explore advanced delivery technologies like nanoformulations to enhance absorption, and use multi‐omics approaches for a more comprehensive understanding of its mechanisms. Investigating how PNL works alongside existing treatments and its efficacy in early disease prevention will be necessary to confirming its safety and effectiveness in patients.

### 3.9 Improving Blood–Brain Barrier (BBB) Penetration and Bioavailability of Paeonol

Despite strong preclinical efficacy, PNL exhibits limited blood–brain barrier permeability and moderate oral bioavailability, which hinder its direct clinical translation for neurological disorders. To overcome these limitations, recent studies have explored several innovative drug delivery strategies. Nanocarrier‐based formulations, including *solid lipid nanoparticles (SLNs)*, *polymeric nanoparticles*, and *liposomes*, have shown promise in enhancing solubility, stability, and brain uptake through improved transcytosis and sustained release [164]. For instance, PNL‐loaded SLNs significantly increased drug concentration in brain tissue compared with free PNL.

Another promising strategy to enhance PNL therapeutic potential is the development of prodrugs, in which PNL is chemically conjugated with hydrophilic or lipid moieties to improve membrane permeability and reduce first‐pass metabolism [165]. These prodrug derivatives demonstrate prolonged systemic circulation and increased efficiency in crossing the blood–brain barrier [166]. In addition, nasal administration offers a non‐invasive route for direct nose‐to‐brain delivery, bypassing hepatic metabolism and efficiently targeting the central nervous system [167]. Formulations such as PNL‐loaded nasal gels and nanoemulsions have shown enhanced brain targeting, faster drug delivery, and improved neuroprotective effects in preclinical animal models. Future research should focus on optimizing these delivery systems, evaluating long‐term safety and efficacy, and translating these strategies into clinical studies to fully realize the neurotherapeutic potential of PNL. These advanced strategies provide valuable frameworks for developing next‐generation PNL formulations with higher therapeutic efficiency, improved pharmacokinetics, and better patient compliance. Future research should focus on optimizing these systems for clinical translation through toxicity assessment, biodistribution studies, and controlled‐release formulations.

Despite the promising neuroprotective effects of PNL demonstrated in numerous experimental studies, several limitations should be considered. First, most of the available evidence originates from in vitro and animal studies, whereas clinical investigations remain limited. Differences in experimental models, treatment durations, dosing regimens, and outcome measures may affect the reproducibility and comparability of findings across studies. In addition, the precise molecular targets and long‐term safety profile of PNL have not yet been fully elucidated. Another important challenge is the pharmacokinetic profile of PNL. Although PNL exhibits favorable biological activities, its moderate bioavailability, rapid metabolism, and limited blood–brain barrier penetration may reduce its therapeutic efficacy in neurological disorders. These limitations highlight the need for advanced drug‐delivery systems, such as nanoparticles, liposomes, solid lipid nanoparticles, and other nanoformulations, to improve stability, bioavailability, and brain‐targeting efficiency. Furthermore, the clinical translation of PNL faces several obstacles, including the lack of standardized formulations, insufficient pharmacokinetic and pharmacodynamic data, and the scarcity of well‐designed randomized controlled trials. Future studies should focus on optimizing formulation strategies, establishing standardized dosing protocols, evaluating long‐term safety, and conducting large‐scale clinical trials to validate the efficacy of PNL in patients with neurodegenerative and neuropsychiatric disorders. Addressing these challenges will be crucial for advancing PNL from experimental research to clinical application.

### 3.10 The Potential of Paeonol in Combination With Other Drugs

Combination therapy refers to the simultaneous use of two or more drugs or treatment strategies to manage a disease more effectively. It enhances therapeutic efficacy, reduces the risk of drug resistance, and minimizes adverse effects. This approach is crucial for treating complex and multifactorial diseases such as cancer, cardiovascular disorders, neurological diseases, HIV, and tuberculosis. PNL has demonstrated significant synergistic potential when combined with other therapeutic agents. Xu et al. (2007) reported that combining PNL with cisplatin enhanced apoptotic induction in human hepatoma cell lines, suggesting its ability to potentiate anticancer effects [168]. Similarly, Li et al. (2012) found that the combination of PNL and danshensu exerted a marked cardioprotective effect against isoproterenol‐induced myocardial injury in rats [169]. These findings suggest that PNL can serve as an effective adjuvant in combination therapy. In the future, exploring PNL‐based combination therapies could lead to improved neuroprotective outcomes, reduced side effects, and the development of multi‐targeted treatment strategies that address the complex nature of neurological disorders.

## 4 Conclusion

PNL stands out as a beneficial natural compound with neuroprotective, antioxidative, and anti‐inflammatory properties, showing efficacy for treating various neurological conditions such as AD, PD, stroke, epilepsy, and mood disorders. Preclinical studies indicate its ability to regulate key pathways like NF‐κB, TLR4, and apoptosis‐related proteins, contributing to improved cognitive and behavioral outcomes. However, there are still difficulties to overcome in turning these findings into real clinical success. PNL's limited ability to cross the blood–brain barrier and the lack of strong clinical evidence create significant challenges. While toxicity studies suggest it is safe at therapeutic doses, inconsistencies in human trials and gaps in understanding its precise mechanisms make further research necessary. To completely get PNL's benefit, innovative drug delivery systems, well‐designed clinical trials, and interdisciplinary research are essential.

## Author Contributions

**Md. Sakib Al Hasan:** conceptualization, writing – original draft, writing – review and editing, project administration, supervision. **Naznin Shahria:** conceptualization, writing – original draft, writing – review and editing, formal analysis, supervision, project administration. **Yasin Emon:** methodology, visualization, writing – original draft, writing – review and editing. **Mohammad Y. Alshahrani:** conceptualization, writing – original draft, writing – review and editing, formal analysis, project administration, supervision. **Emon Mia:** investigation, methodology, software, formal analysis, validation. **Faisal H. Altemani:** conceptualization, writing – original draft, writing – review and editing, visualization, software, resources. **Rituparna Biswas Suma:** investigation, visualization, software, resources. **Umme Habiba Sumaya:** software, resources, data curation. **Mohammed Burhan Uddin:** writing – original draft, visualization, software. **Md. Torequl Islam:** conceptualization, writing – original draft, investigation, writing – review and editing, visualization, validation, methodology, software, formal analysis, project administration, resources, supervision, data curation.

## Funding

The authors have nothing to report.

## Conflicts of Interest

The authors declare no conflicts of interest.

## Data Availability

Data sharing is not applicable to this article, as no new data were created or analyzed in this study.
